# A Role for Photobiomodulation in the Prevention of Myocardial Ischemic Reperfusion Injury: A Systematic Review and Potential Molecular Mechanisms

**DOI:** 10.1038/srep42386

**Published:** 2017-02-09

**Authors:** Ann Liebert, Andrew Krause, Neil Goonetilleke, Brian Bicknell, Hosen Kiat

**Affiliations:** 1 Australasian Research Institute, Wahroonga, Australia; 2 Sydney University, Sydney, Australia; 3 Maitland Hospital, Maitland, Australia; 4 Blacktown Hospital, Sydney, Australia; 5 Australian Catholic University, North Sydney, Australia; 6 University of New South Wales, Kensington, Australia; 7 Macquarie University, Marsfield, Australia

## Abstract

Myocardial ischemia reperfusion injury is a negative pathophysiological event that may result in cardiac cell apoptosis and is a result of coronary revascularization and cardiac intervention procedures. The resulting loss of cardiomyocyte cells and the formation of scar tissue, leads to impaired heart function, a major prognostic determinant of long-term cardiac outcomes. Photobiomodulation is a novel cardiac intervention that has displayed therapeutic effects in reducing myocardial ischemia reperfusion related myocardial injury in animal models. A growing body of evidence supporting the use of photobiomodulation in myocardial infarct models has implicated multiple molecular interactions. A systematic review was conducted to identify the strength of the evidence for the therapeutic effect of photobiomodulation and to summarise the current evidence as to its mechanisms. Photobiomodulation in animal models showed consistently positive effects over a range of wavelengths and application parameters, with reductions in total infarct size (up to 76%), decreases in inflammation and scarring, and increases in tissue repair. Multiple molecular pathways were identified, including modulation of inflammatory cytokines, signalling molecules, transcription factors, enzymes and antioxidants. Current evidence regarding the use of photobiomodulation in acute and planned cardiac intervention is at an early stage but is sufficient to inform on clinical trials.

Heart failure is an increasing health burden worldwide, with myocardial infarct (MI) size suggested as the major determinant of adverse outcomes^1^. Initial cardiomyocyte death due to ischemic conditions is followed by subsequent apoptosis and myocardial dysfunction, instigated by the reperfusion of areas devoid of blood flow. Post infarction remodelling along with the incurred cardiomyocyte death, results in reduced contractility, excessive left ventricular chamber dilation, infarct related wall thinning, compensatory hypertrophy of non-infarcted regions and increased deposition of fibrillar collagen^1^,^2^. Although interventions targeted at myocardial ischemic-reperfusion (MIR) insult have become less invasive and more effective in reducing mortality, ongoing management of morbidity among survivors is a substantial challenge^1^.

When the myocardium no longer receives oxygenated blood from the compromised coronary vessel the “area at risk” is rendered ischemic and subject to distinct metabolic processes that result in necrosis and cell death^1^,^3^. Under ischemic conditions, oxidative phosphorylation ceases, which reduces mitochondrial membrane potential and the availability of cellular ATP. This causes the engagement of anaerobic glycolysis, which in turn induces an influx of Na^+^ through the Na^+^/H^+^ exchanger. Mitochondrial Na^+^ levels are further exacerbated by the reduction of Na^+^/K^+^ ATPase, which requires ATP for activation. In an attempt to restore cellular pH, Na^+^ is removed by the 2Na^+^-Ca^2+^ ion exchanger, causing a substantial influx of Ca^2+^ 3,4. Accumulated mitochondrial Ca^2+^ remains within the mitochondria while mitochondrial permeability transition pores (MPTP), which are dependent on intracellular pH levels, remain closed^5^.

When blood flow is restored, reperfusion causes additional injury, thought to be related to the surge of oxygen return. Yellon and Hausenloy^6^ have suggested that reperfusion alone can contribute up to 30% to 40% of total infarct size following coronary artery occlusion. Microvascular obstruction has been suggested to cause damage during coronary events, however its contribution to infarct size, if any, remains unclear^4^. Under conditions of rapid re-oxygenation, the shift in ionic flux results in restoration of cellular pH and the rapid alteration of cellular pH, rather than the return of oxygen, may be the stimulus that activates processes leading to cell death^5^. A speed dependant relationship of pH restoration has been identified, where the intensity at which oxygen returns determines the amount of reactive oxygen species (ROS) released and the opening of the MPTP, allowing accumulated Ca^2+^ into the cytoplasm. Disordered intracellular Ca^2+^/ROS balance ultimately leads to dysregulation of the MPTP and rupture of the sarcolemma^5^,^7^,^8^. A more severe form of apoptosis is oncosis, where cell death is characterised by cell swelling and karyolysis during MIR injury. Factors that mitigate against oncosis include the presence of melatonin^9^.

Secondary damage as a result of the innate immune response has been suggested as an immediate and delayed process that also contributes to infarct size. Persistent inflammation has been identified as harmful, preventing infarct repair^2^. However, the exact involvement of inflammation has yet to be fully elucidated^4^. Neutrophils, monocytes, and macrophages, which are responsible for remodelling and removal of dead or dying tissue, depend on specific spatiotemporal and quantitative signalling for their activation^10^. Modulation of these signalling processes, especially in the acute vulnerable period following MIR injury, presents another pathway for reducing infarct size.

MIR injury is also associated with potentially serious systemic effects, such as increased morbidity, increased mortality (9%) at one year^11^ and neurological impairment, where it is estimated that only 10% of resuscitated patients are neurologically intact when discharged from hospital^7^. MIR injury as a sequela of cardiac surgery, may also lead to a greater rate of post-operative cognitive dysfunction (POCD) than other types of surgery^12^. Thus recovery from MI is the gold standard for effectiveness of treatments that precondition against MIR injury.

While there are many therapies to reduce the effect of ischemia, there has been less success in treating reperfusion injury, although a number of novel potential therapies have been proposed^1^,^11^,^13^. These include ischemic preconditioning, where brief episodes of ischemia followed by reperfusion, are introduced before a sustained ischemia. Ischemic preconditioning has been shown to reduce infarct size substantially (30–80%) and can last for 2 to 3 hours after the preconditioning event, as well as having a second window of protection that occurs 24 hours after preconditioning and can last for about 48 hours^14^. Ischemic post-conditioning, where the rate of reperfusion is slowed by short episodes of myocardial ischemia (using, for example, an angioplasty balloon) may also be effective in reducing MIR injury^14^. Remote ischemic conditioning, where brief non-lethal episodes of ischemia and reperfusion are applied to an area remote from the heart, such as an arm or leg, has also been shown to have some effect on MI size^6^. While ischemic conditioning has been demonstrated in animal models, there has been less success in the translation of this therapy to clinical trials^15^,^16^, although balloon angioplasty post-conditioning and hypothermia have shown some positive effects^17^. Ischemic preconditioning is also limited to conditions where the potential for MIR insult can be predicted, such as coronary artery bypass grafts (CABG) surgery, and ischemic post-conditioning is limited by the very short window (5 to 10 minutes) during which the treatment can be effective^14^. In addition to ischemic conditioning, there are a number of experimental pharmaceutical products with the potential to reduce MI size. Alpha-melanocyte-stimulating hormone (α-MSH) has shown some promise^18^, the use of volatile anaesthetics for cardioprotection during open heart surgery can produce modest effects^19^ and the use of cyclosporin A^20^ and exenatide^21^ have been shown to produce reductions in MI size. The disadvantage of drug and anaesthetic intervention is the potential to introduce serious side effects, complicated by the proteostasis of the patient, as well as disease (e.g., diabetes) and interactions with other drugs^22^. These side effects can potentially be as severe as Parkinson’s disease and Huntington’s disease-like symptoms^7^.

In short, MIR injury involves a complex redox stress response, for which there is so far no effective treatment, although a number of novel treatments have been proposed. A goal for such treatment would be a mechanism to switch between the deleterious redox stress reactions, towards protective redox conditions. The complexity, however, of the switching mechanism has hindered an effective therapeutic regime^23^. A potential target for treatment intervention is the modification of the mitochondrial response to oxidative stress.

Photobiomodulation (PBM) is the low power (1–500 mW) non-thermal delivery of photons in the visible or near infrared spectrum (405–1000 nm) that elicits a beneficial biological response in cells and tissues^24^. PBM can include light emitting diodes (LED) and low-level laser therapy (LLLT). Phototherapy has a long history of application in medicine. The earliest scientific report was the use of red light in the treatment of smallpox scars by Neil Finsen in 1903^25^ and his use of ultraviolet light for the treatment of lupus vulgaris, which resulted in a Nobel Prize in 1903. Laser light was used in radiation ulcer attenuation after the Chernobyl nuclear accident^26^ and light has been used as a treatment for bilirubin dysfunction in neonates for many decades^27^. Interestingly, when light is used for premature neonate jaundice treatment, there is a concomitant effect in patent ductus arteriosus, where the systemic effect of the light application can cause cardiac vasodilation effects and prevents the closure of the patent ductus, which can be avoided by the use of light impermeable chest shielding^28^. Recently 670 nm light has been proposed as a treatment for oxygen-induced retinal disease in neonates^29^. PBM has been increasingly used for treatment of ulcers^30^, wounds^31^, neuro-inflammation^32^,^33^, pain^34^, lympoedema^35^, macular degeneration^36^ and tendon healing^37^. The use of LLLT in various clinical applications has been reviewed by Chow *et al*.^34^ in the area of chronic neck pain, by Khan and Arany^38^ for wound healing, by Geneva *et al*.^39^ for retinal disease, by Agrawal *et al*.^40^ as a preconditioning treatment for various diseases and by Carlos *et al*.^41^ for cardiac remodeling after myocardial infarction. Animal models have also suggested a role for the PBM treatment of neurodegenerative diseases^42^, such as Alzheimer’s disease^43^, Parkinson’s disease^44^, multiple sclerosis^45^ and as a preconditioning treatment against post-operative cognitive dysfunction^46^. Natural light sources have also been shown to be important in recovery from spinal surgery^47^ and management of cardiac risk patients^48^. An emerging body of experimental evidence as well as some clinical trials support the application of PBM in conjunction with routine cardiac interventions, which warrants a systematic review of PBM application and MIR injury.

The aim of this contribution is to report the results of a systematic review into the experimental evidence in tissue studies, animal studies and clinical trials for the use of PBM in the treatment, intervention and management of myocardial reperfusion injury, and to summarize the underlying mechanisms and metabolic signalling pathways found to underpin this effect. The results of the review can be used to inform scientists and clinicians on the potential scope of further experimental studies and clinical trials, the creation of best practice guidelines and the use of PBM in other areas of cardiovascular intervention.

## Results and Discussion

### Quality and Limitations of Studies

**Figure 1:**
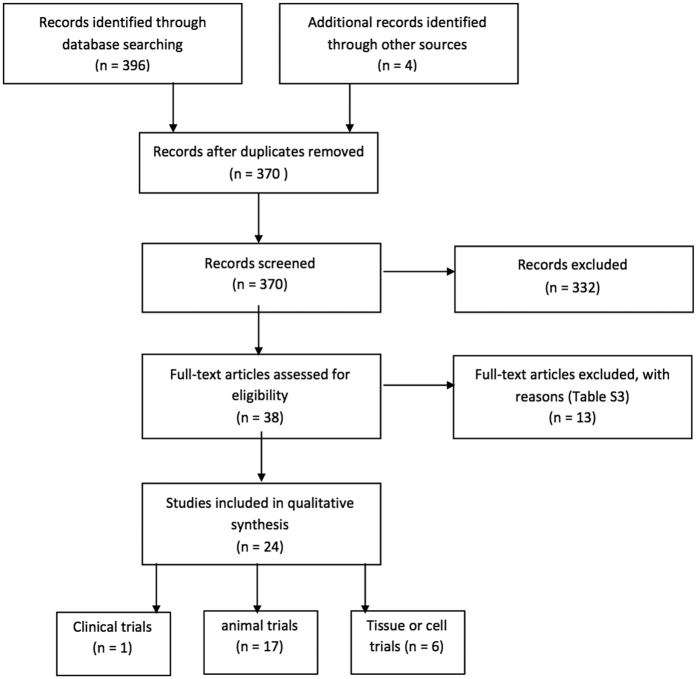
Flow diagram of selection of articles, based on PRISM guidelines ( **http://www.prisma-statement.org**).

**Figure 2:**
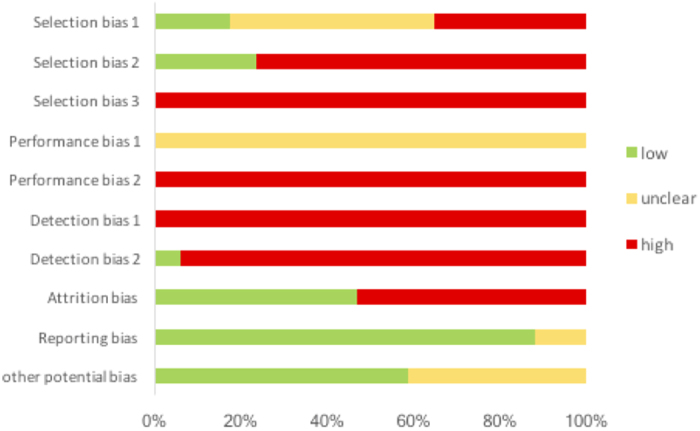
Risk of bias score for each risk item in animal studies, as assessed using the SYRCLE tool^50^.

The review of articles was conducted according to PRISMA guidelines (http://www.prisma-statement.org) (Fig. 1). Since most of the articles reviewed pertain to animal or tissue experiments, with only one clinical trial, the extent to which results can be extended to human effects is necessarily limited. In the single clinical trial, assessment using the JADAD method^49^ resulted in a score of 4, indicating high quality. The use of the SYRCLE risk of bias tool^50^ to assess quality of animal studies indicated a high or unknown risk of bias for most studies in the majority of categories (Table 1 and Fig. 2). Only two categories were assessed as having a low risk of bias for the majority of studies. While reporting bias (item 9, Table 1) was assessed as low, the possibility of publication bias, in that results have been selectively reported, cannot be discounted, especially as no adverse effects or negative findings of the effect of PBM were described. It should also be kept in mind that it is difficult to translate results of animal studies to humans, due in part to the variations in physiology and anatomy between the species (for example, rats have fewer collateral coronary arteries than dogs and humans).

### Characteristics of Studies

Experimental designs in the studies reviewed ranged from *in vitro* cardiomyocyte cultures to a single human clinical trial. Of the 23 studies included, 17 (74%) were conducted using animal models (13 on rats, 2 on mice, 1 on rabbits and 1 on dogs and rats), 2 were conducted on isolated rat hearts, 3 on tissues or cell cultures and 1 was a clinical trial (Table 2). The application site for PBM in animal studies varied with experimental design, with 6 studies using trans-thoracic application of PBM (with skin shaved or removed), 12 irradiating the myocardium directly and 3 studies irradiating sites distal to the heart (leg muscle or tibia). In the clinical trial there was a transthoracic administration of PBM. Studies on isolated tissue and cell samples involved irradiation without direct contact. PBM was administered as preconditioning treatment, as post-conditioning treatment, as treatment during surgery or reperfusion, or as a combination of treatments. Total intervention dose ranged from 0.6 J to 36 J, with a mean of 6.26 J. All trials used wavelengths between 630 nm and 830 nm, with the human trial using infrared light (810 nm) and the majority of animal trials used red light (at around 660 nm). All animal studies reported physical or histological outcomes after PBM intervention and 18 reported molecular changes.

### Infarct Size Reduction, Histological Profile and Long-Term Effects

The most significant finding from this review was the positive effect of PBM on modulating infarct size and the improvement of cardiac remodelling^51^,^52^,^53^,^54^,^55^,^56^,^57^,^58^,^59^,^60^,^61^,^62^,^63^. These results were achieved using a range of experimental designs, wavelengths and dosages. Three studies^59^,^60^,^61^ reported a reduction in total infarct size of greater than 60% with one^60^ reporting MI size reduction of 76% when irradiation was applied to the tibia of the rat (remote preconditioning), compared to a 31% reduction when applied locally to myocardium. Yaakobi *et al*.^58^ reported consistently reduced infarct size compared to controls throughout a 45-day follow-up period. The results agreed with other studies that have shown PBM can significantly decrease the narrowing of coronary arteries, decrease rates of restenosis after stenting procedures^64^,^65^,^66^ and is a therapeutic option for severe medically refractory angina^67^ and healing of sternotomy incisions^68^.

Histological analysis in a number of studies revealed improvements in cardiomyocyte arrangement^51^,^53^,^58^,^69^, including reductions in dense collagen, reduced ventricular wall thinning and increased mesenchymal and cardiac stem cells. Gavish *et al*.^70^ also reported that PBM modulated collagen synthesis in a porcine aorta model, along with changes to the levels of matrix metalloproteinase-2 (MMP-2), a regulator of collagen synthesis.

### PBM Dose, Timing and Treatment Regime

All studies showed the safety of PBM in animal and human subjects, including direct myocardial irradiation and the clinical trial did not report any side effects or adverse reactions in patient populations as a result of PBM treatment. Both LLLT and LED were shown to be effective as a source of PBM and all wavelengths tested showed positive results, including red and infrared wavelengths (Table 2). As yet there have been no studies on super-pulsed laser infrared (904–980 nm) wavelengths. The reported effects of PBM on MIR injury were dose dependant and mirrored PBM effects that have been reported in the literature for treatment of conditions such as chronic pain, neurodegenerative disease, lymphoedema and macular degeneration^34^,^35^,^36^,^42^.

There appears to be wide windows for the dose and timing of PBM treatment. In a number of studies, PBM was shown to be effective both as a preconditioning treatment and if applied during reperfusion or in the immediate post (4 hour) surgical window. There was also good evidence^60^,^71^ that PBM administered to a distal area (tibia or leg muscle) produced a positive effect on infarct size. This is consistent with the abscopal effect seen in application of PBM^72^ with, for example, neuroprotection where targeting remote tissues with PBM can decrease the symptoms of Parkinson’s disease^73^. This abscopal or systemic effect has been attributed to the downregulation of pro-inflammatory cytokines and upregulation of anti-inflammatory cytokines^73^,^74^ as well as to the proliferation of mesenchymal stem cells^59^,^60^,^75^, both of which PBM is known to influence^76^. It may also possibly be due to a systemic mitokine response (see below).

One study found that a higher PBM dose was more effective in the initial phase of MIR to reduce injury^58^, 804 nm LLLT at 12 mW/cm^2^ having more effective cardioprotection than 6 mW/cm^2^. This is in contrast to the most effective dose given 1.5 hour after MIR being a combination of 12 mW/cm^2^ and 5 mW/cm^2^, which was found to be superior to two doses of 12 mW/cm^2^, leading to the conclusion that LLLT may be less beneficial when given too frequently. The power density required to achieve a reduction in MI size was similar for rats, dogs and humans. For infrared 803–810 nm the dose window indicated is 5 to 15 mW/cm^2^ (with 25 mW/cm^2^ less effective)^53^,^77^, which equates to 1 to 6 J/cm^2^ for rats^58^ and 6 J/cm^2^ for the human trial^78^. This is consistent with other studies of treatment with PBM that have demonstrated a biphasic dose response, where a therapeutic effect is achieved with an optimal dose within a wide dose window, outside of which there is no effect^79^. Oron *et al*.^53^ stressed that the complex sequential physiological processes after MIR injury required different doses of PBM at different stages of reperfusion. PBM administered after the reperfusion event (post-conditioning) was found to be effective in a number of studies, even after a considerable delay. In fact, when a delayed post-conditioning treatment was omitted from the treatment regime, the effect on MI size was reduced^58^. Evidence for treatment protocols from a range of animal studies and the clinical trial would seem to suggest that a combination of preconditioning, immediate and post-application (with red or infrared wavelengths of LED or Laser) produce the most positive effects. All of these dose and timing factors would be important for administration of PBM in any planned clinical trial.

The achievement of a therapeutic dose in small experimental animals is not in question using near infrared wavelengths^80^, however there is the possibility that transthoracic PBM may not reach a therapeutic dose in cardiac tissue in humans. While the transmission of light to the heart does not appear in the literature per se, it can be inferred from other studies. For example, the use of PBM to treat traumatic brain injury has included studies that have demonstrated that transcranial LLLT can reach therapeutic doses 40 to 50 cm deep with infrared irradiation (800–830 nm), using head simulations^81^, formalin fixed^82^ and unfixed human cadaver heads^83^. The average parasternal, skin-to-heart distance has been reported as 32.1 ± 7.9 mm^84^. In addition, in the clinical trial reviewed here, the transthoracic application of PBM resulted in changes to cardiac and inflammation markers^78^.

### Mitochondrial Effects

The overall trend from the studies was a positive effect on mitochondrial respiration pathways, including an increase in the availability of ATP and nitric oxide (NO). Zhu *et al*.^85^ noted 15% higher ATP levels in isolated hearts during induced cardioplegia when treated with PBM and Oron *et al*.^53^ showed a 21% reduction in mitochondrial damage in an animal model, which, it was suggested, may account for sustained ATP production.

**Figure 3:**
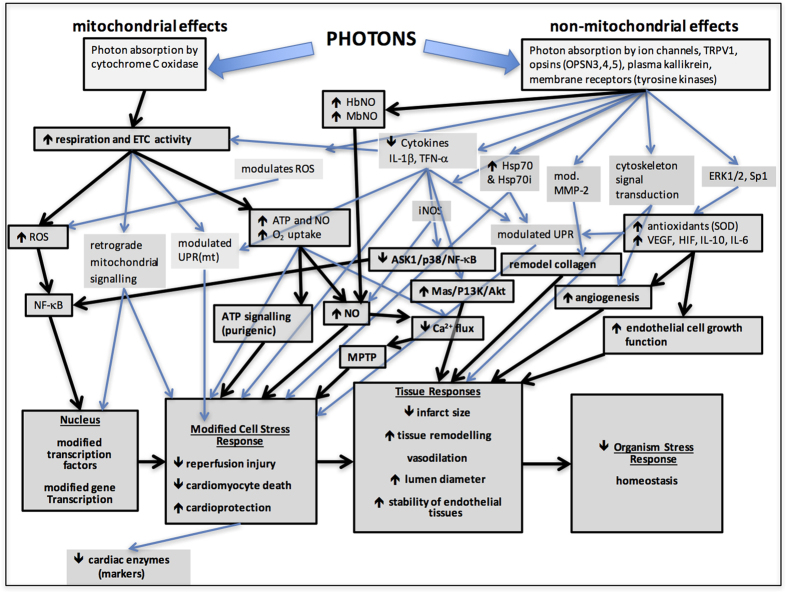
Cell signal transduction pathways identified in the reviewed papers as being modified by photobiomodulation in tissue, animal and human models of cardiovascular damage. Key pathways are shown as darker lines.

One of the major photon receptors is generally believed to be the cytochrome C oxidase (COX) enzyme in the electron transport chain and stimulation of this enzyme sets in motion multiple cascading signal transduction pathways^86^. Heart tissue, however, responds differently from other tissues to mitochondrial COX signalling pathways, due to the tissue specific expression of signalling molecules^7^, which might make heart tissue more susceptible to ischemic and reperfusion injury than other tissues. The response of the mitochondria to reperfusion is a critical factor in the health or otherwise of myocardial cells and tissues. Protection of the mitochondria, particularly the MPTP, has been called “the Holy Grail” of cardioprotection^13^ and phosphorylation events that regulate COX are ideal targets for therapeutic interventions^7^. The effect of PBM on COX is generally accepted^2^ and is believed to be a major reason for the effect PBM on mitochondrial health (Fig. 3), due to the increased production of ATP and the activation of ATP dependent ion pumps^86^,^87^. Recently however, it has been demonstrated that near IR has no effect on isolated COX protein^88^, which may suggest a more complex interaction between light and the mitochondria, possibly an indirect effect. This was also implied in a study of retinopathy in diabetic mice^89^ and in diabetic rats^90^, where there was no evidence found for a PBM effect on mitochondria. Never-the-less, an effect of PBM on the mitochondria was suggested by a number of the studies reviewed here (Fig. 3), with a positive effect of PBM on both mitochondrial respiration and mitochondrial retrograde signalling and major impacts on the regulation of Ca^2+^ ion channel flux, NO production through COX and increased in ATP levels. These factors ensure the maintenance of mitochondrial retrograde signalling, which has been identified as a regulator of many cellular activities in both normal and pathological states^91^, including mitochondrial retrograde signalling in MIR^92^ and the ischemic preconditioning effect of cardioprotection^40^. Mitochondrial homeostasis is important in the cell stress response^93^, which can be regulated by the level of cellular melatonin in the MIR injury response, where elevated melatonin is neuroprotective against myocardial cell oncosis^9^. Modulation of the mitochondrial retrograde signalling response would also have an effect on systemic metabolism through the mitochondrial unfolded protein response (UPRmt)^94^,^95^. Since proteomic stress can induce changes in redox stress across tissues, this may be important in the limitation of cardiomyocyte death and scar formation^96^ as well as in organism wide neuroprotection. This may involve a mitokine response^95^, which could also have implications in preconditioning against ischemic reperfusion injury. The modulation of the UPRmt would restore homeostasis through mitochondrial hormesis (mitohormesis) (see Fig. 3).

### Non-Mitochondrial Signalling by PBM

Non-mitochondrial effects of PBM involve signalling pathways that originate from photon absorption and protein conformational modulation at the cell membrane (Fig. 3). Photons can be absorbed by ion channels (including TRPV1, K^+^ and Ca^2+^ channels), membrane receptors including tyrosine kinases and a variety of photoactive molecules described as opsins^38^. Downstream signal transduction is known to affect inflammatory cytokines^97^, heat shock proteins^98^, endothelial and axonal cytoskeleton morphology^99^,^100^, antioxidant IL10^101^, growth factors vascular epithelial growth factor (VEGF) and MMP-2^102^, inducible nitrite oxide synthase (iNOS)^103^, and superoxide dismutase (SOD)^104^ as well as modifying ROS and NO. These have a variety of effects, such as improving tissue responses, including cardiac tissue, endothelial tissue and arterial lumen diameter.

An example of a signalling pathway modified by PBM in MIR is the suppression of the ASK1/p38/NF-κB signalling pathway^71^, which is important in cardiomyocyte necrosis following myocardial infarction^2^ and which has also been shown, in an *in vitro* model of wound healing, to enhance tissue regeneration and angiogenesis^105^. A pathway that was not monitored in any study reviewed here is the Akt/GSK-3β pathway^106^, involved in neuronal survival in traumatic brain injury. This pathway has been demonstrated to be modulated by PBM and to be neuroprotective against apoptosis induced by amyloid β peptide^107^. This pathway might be predicted to also be involved in protection against MIR injury by PBM.

PBM was shown in a number of the studies reviewed here to influence a variety of non-mitochondrial signal transduction pathways, which are linked to a decreased cell adverse stress response, improved tissue responses (decreased MI size, reduced restenosis, etc.) and a restoration of homeostasis (see Fig. 3). While these modulations of cell signalling can also be achieved using drug and anaesthetic strategies^5^, these treatments have the potential for serious side effects, as previously noted.

### Redox State and Antioxidants

In addition to the respiratory chain, NO is also produced in a number of reactions outside of the mitochondria. A number of studies reviewed here demonstrated an increase in NO following PBM treatment, as well as increased levels of iNOS and endothelial nitric oxide synthase (eNOS). Tuby *et al*.^77^ reported a significant elevation in iNOS, which increased with increasing PBM dose. The increase in iNOS levels was recorded after 2.5 hours and peaked at 2 days, which indicated an ongoing role for NO after the acute insult. Interestingly, enhanced levels of iNOS were also observed in non-infarcted but irradiated myocardium, which raises questions concerning the modulating effect of PBM on this enzyme in non-traumatic pathological states. Manchini *et al*.^63^ however reported reductions in iNOS in the intervention group, 3 days post injury, although plasma nitrite and nitrate concentrations (NOx) were markedly higher, indicating a transient effect of PBM on NO levels.

In one study^62^, NO was shown to be released from nitrosyl heme proteins in the irradiation group and two other studies^54^,^55^ reported an increase in NO from MbNO and HbNO, independent of nitric oxide synthase.

NO is important in the PBM myocardial protection during ischemic reperfusion injury, both in the cell stress response and also in regulating Ca^2+^ homeostasis^108^ and thus limiting the effect of mitochondrial Ca^2+^ overload from MPTP. NO signalling may also lead to mitochondrial S-nitrosation, which slows reactivation of mitochondrial metabolism at the onset of re-oxygenation^7^. This slowing of reactivation in post-conditioning may be particularly important to mitigate against further MIR injury. Non-NOS sources of NO have been argued to be a major mechanism behind PBM protection against MIR injury^55^.

Increased levels of a number of anti-oxidants and ROS were reported after PBM intervention. These included creatine phosphokinase (CPK)^78^, lactate dehydrogenase (LDH)^78^, dichlorofluorescein (DCFH)^109^, glutathione (GSH)^98^, SOD^110^,^111^,^112^ and interleukin 10 (IL-10)^57^,^71^,^113^,^114^.

While two studies reported that levels of SOD increased within 1 hour post irradiation^110^,^111^, others^109^,^115^ reported a reduction in SOD activity in PBM treated groups, when irradiation occurred 4 and 3 weeks after rats were exposed to ischemic insult. These results suggest that PBM induces an acute transient increase in SOD activity immediately after MIR injury. This was confirmed in an experiment where SOD activity was shown to double when isolated rat hearts were irradiated immediately after MIR injury^112^. Interestingly Malinovaskaya *et al*. found that while non-coherent light resulted in an increase in SOD, laser brought about a decrease in SOD activity^111^.

Yaakobi *et al*.^58^ found a 2.2-fold increase of heat shock protein inducible factor (Hsp70i), 5 hours post irradiation using 810 nm. Zhang *et al*.^110^ reported increased levels of gastrin-releasing peptide (GRP) 78 (a chaperone to Hsp70) at 1 hour, 1 day and 1 week, indicating a sustained up-regulation of this protein in the later stages of the tissue recovery cycle. Heat shock proteins have a role in mitochondrial membrane homeostasis and have an important role in protein folding, unfolding and the cell stress response^116^. Hsp70 is also an important factor in the amelioration of acute lung injury induced by gut ischemia in the rat model, where PBM up-regulates peroxisome proliferator-activated receptor-γ (PPARγ), which in turn results in an increase in Hsp70 production and a decrease in inflammation and lung injury^85^.

### Inflammatory Cytokines

Inflammatory cytokines regulate the cellular stress response and the inflammatory cascade associated with the ischemic event that causes injury^23^. The balance between pro and anti-inflammatory cytokines is important in determining prognosis after MIR injury, especially the balance in redox signalling molecules, which appears to act as a switch between protective oxidative signalling and damaging oxidative signalling^23^. While this switching mechanism is not yet fully understood, it appears that PBM is able to affect the switching in a positive way, resulting in cardioprotection. This was consistent across all studies and parallels the PBM effect on cytokines in non-cardiac studies, including macular degeneration^36^, wound healing^38^, muscle pre-conditioning^40^, gut ischemia and reperfusion^98^ and neuroprotection against Alzheimer’s and Parkinson’s diseases^42^ and, potentially, POCD^46^.

Six studies reported changes in levels of the inflammatory markers IL-1α, 1β, 2, 4, 6 and 8 after PBM^57^,^63^,^71^,^114^. In a human clinical trial with patients undergoing angioplasty and cardiac stenting (excluded from this review), Derkacz *et al*.^114^ also found that IL-1β, and IL6 were reduced when irradiated with 808 nm with a total treatment dose of 9 J/cm^2^. In contrast to these findings, Manchini *et al*.^63^ found strong increases in IL6 in both control and PBM groups (660 nm and 22.5 J/cm^2^) three days post intervention. In a comparable study, Hentschke *et al*.^71^ showed that a four-week delay in PBM application resulted in reduced IL6 levels in the group treated with 660 nm 21 J/cm^2^ but significantly higher IL6 levels in the group treated with 3 J/cm^2^. Together these results suggest that there was a dose window and a dose dependant relationship between PBM and levels of IL6.

In the clinical trial reviewed here^78^, repeated doses of 808 nm PBM resulted in reduced white blood cell, lymphocyte and neutrophil activity 5 days post cardiac artery bypass graft (CABG) surgery. In a rat reperfusion injury model^98^ it was reported that an acute increase in myeloperoxidase (MPO) occurred after irradiation with 660 nm laser, indicating an increase in neutrophil granulocyte activity. Manchini *et al*.^63^ found that kinin B2 receptor mRNA expression and Mas receptor protein expression was increased after MI and PBM, whereas PBM significantly decreased the kinin B1 and significantly reduced angiotensin-converting enzyme (ACE) mRNA expression, all of which affect vasodilation.

### Growth Factor Modulation

Three studies showed significant increases in expression of VEGF after PBM. Tuby *et al*.^77^ demonstrated significant increases in expression as early as 2.5 hours after treatment, which continued to 24 and 48 hours, reverting back to pre-treatment levels by 72 hours. There was also a dose dependent relationship, with power densities 5, 12, and 17 mW/cm^2^ increasing expression rates by 2, 2.3, and 1.3 fold respectively. Zhang *et al*.^110^ also found significant increases in VEGF 1 hour and 1 day post intervention, with no significant difference in levels after 1 week. This is consistent with other studies investigating the mechanisms of PBM action^102^. The increase in VEGF promotes the proliferation of endothelial cells and angiogenesis, important in the cell stress response under hypoxic conditions^117^ or under high concentrations of ROS^94^. VEGF is also important in the homeostatic control of the unfolded protein response in the endoplasmic reticulum and in the mitochondria^94^. In addition, PBM has an indirect action on mesenchymal stem cell proliferation^60^,^69^ and the interaction between mesenchymal stem cells and VEGF mRNA modulates cell adhesion and proliferation in a nutritionally deprived model^118^.

### Cardiac Markers

There was a reduction in cardiac enzyme marker troponin in the rat model^52^ and creatine phosphokinase (CPK) in the rat^52^ and the human study^78^. These are clinically important markers for cardiac damage, which may be important in the design of PBM trials for the evaluation of the success of dose and timing of PBM protocols.

### Cytoskeleton Modulation by PBM

In a study on nutrient stressed rabbit aortic endothelial cells^99^, it was shown that irradiation with laser at 685 nm at 8 J/cm^2^ for 7 days caused reorganisation of actin filament stress fibres and filament proliferation, such that the cell regained its structure to a pre-stressed state. This may be a similar mechanism to the reorganization of the cytoskeleton seen after PBM application to dorsal root ganglion nerves in pain blockade^46^,^100^; a process strikingly similar to the cytoskeleton remodulation in the neuroprotective mechanism of rapid ischemic tolerance defence against NMDA excitotoxicity. These mechanisms have been reviewed by Liebert *et al*.^46^. This rapid ischemic tolerance mechanism may be relevant to the PBM effect in the animal model for infarct size reduction in MIR. It is often observed that rapid ischemic tolerance induce by PBM (such as in pain blockade) requires a higher dose in the initial stages, which is also the case in cardioprotection^77^, suggesting a similar mechanism.

## Conclusion

Cardiac protection, both in clinical use and preconditioning applications, will become progressively more important with increasing numbers of cardiac procedures in the future and the further burden of cardiac disease, evident in the incidence of morbidity and potential mortality and the escalating cost of treatment. The evidence outlined in this review from a range of *in vitro* and *in vivo* animal studies, as well as one clinical trial, suggests that PBM may have a role as a cardioprotective agent against MIR injury and could protect against the initial cardiac ischemic event and the ongoing damage caused by reperfusion. PBM has been shown to affect a variety of signal transduction pathways that are critical to switching from the deleterious redox stress reactions that occur as a result of reperfusion, towards the more protective redox conditions that can limit injury and promote repair. This could ultimately lead to improved tissue responses, including reduced infarct size and lower rates of restenosis. PBM is non-invasive, simple to administer, inexpensive and has no known side effects, unlike other interventions that appear to have limited evidence of efficacy and potentially deleterious side effects. PBM could therefore be considered as a potential alternative to drug and anaesthetic pre-treatments for MIR injury. It also appears to be an effective post-conditioning therapeutic protocol, thus extending the available time for intervention to treat MIR injury.

The number of well-designed clinical trials is limited (one in this review), but the available evidence from animal and tissue studies suggest that further clinical trials are warranted. The timing and dosage of PBM appears to be complex, with different doses required at different stages of reperfusion, in order to achieve optimal treatment outcomes; something that must be considered when planning clinical trials. The cardiac markers of troponin, CPK and other novel biochemical indices such as osteopontin^119^ could be used as markers of MIR injury in these future trials. Evidence would suggest that a combination of preconditioning, immediate and post application treatment would be appropriate, with a potential role for remote application of PBM to produce a cardioprotective preconditioning abscopal effect.

## Methods

### Search Strategy

This review was conducted according to PRISMA guidelines and the overall search strategy is shown in Fig. 1. The search of published articles was conducted on the 28^th^ of August 2015. PubMed, Ovid (OvidMedline), Scopus, and Web of Science, journal databases were searched with restrictions set to 1995-current, and English publications only (see Supplementary Table 1). Keywords used for the search included “laser, low level” and was combined with “myocardial”. Initial searches were expanded by adding derivatives: “ischemia”; “infarct”; “rupture”; “arrest”; and “failure”. Preliminary search results indicated that certain keywords such as “cardiovascular” and “disease” returned an excessive number of results when searching databases. These keywords were not used in order to limit the number of irrelevant papers captured during the initial search, which may have meant that studies that included these keywords were overlooked. Duplicate articles from the database search results were removed. Titles and abstracts of each paper were screened, and irrelevant articles removed. The reference lists of relevant papers were searched for additional articles, which were then added to the list of articles.

### Eligibility Criteria

Once all full-text copies were obtained, each article was submitted to the eligibility criteria set out by authors (Supplementary Table 2). Articles investigating therapeutic, procedural or methodological applications of PBM were accepted, including application of PBM as an adjunct to surgery, or with standard medical management in a clinical population. The application of the irradiation could be to any part of the body. Due to the small number of clinical trials available, research conducted using animal models, isolated tissues and cell cultures were also accepted. Experimental design had to be representative of either ischemic and/or reperfusion injury. The primary outcome of the review was changes to mortality, cardiac tissues or cells or a change to molecular markers of cardiac function. Secondary outcomes were changes to other molecular markers, such as signalling molecules, redox markers or cytokines. All studies had to report a minimum of wavelength, power output and dose intervention parameters, or the missing parameter had to be calculable using alternate parameters, such as fluency and power density. Studies using combination therapies were omitted to ensure clarity when collating treatment effects. Populations with diseases or co-morbidities, with the exception of those directly involving MIR pathophysiological processes, were excluded to ensure conformity of examined population. Likewise, investigations involving organisms or cell structures with atypical genetics, or those containing disease-causing microorganisms were excluded to avoid false negative results.

### Language and Time Restrictions

Language was restricted to English. Preliminary searches returned 21 articles not available in English. Titles of these papers were screened and a further 12 papers identified as potentially relevant. A sample of these were selected and translated into English. Upon review by AL, it was deemed that these articles were not suitable for inclusion. Examination of the publication trend indicated an increase in article number after 2004, with 50% of included articles published since 2010. Articles were therefore limited to those published after 1995. While the decision to only review articles after 1995 limited the number of articles captured, it is highly likely that key insights and findings from before 1995 would have been re-examined in later articles.

### Qualitative assessment

Twelve articles did not meet the inclusion criteria. These articles are shown, along with reasons for exclusion, in Supplementary Table 3. The remaining studies were then divided between AK, AL and NG for data extraction. The experimental design, including population studied, number, outcome measures, treatment protocol, and laser dose parameters were recorded. All recorded data was collated into results tables and synthesized (BB and AK) to identify analogous results. Laser dose parameters were standardized by conversion into total dose administered, using the formula from Bjordal *et al*.^120^. Qualitative analysis of the clinical trial was conducted using the method of Jadad & McQuay^49^. Risk of bias for animal studies was assessed using the SYRCLE tool of Hooijmans *et al*.^50^. Interpretation of the articles in this review is necessarily descriptive, due to the limited number but wide range of studies that have investigated the effect of PBM in MIR injury.

## Additional Information

**How to cite this article:** Liebert, A. *et al*. A Role for Photobiomodulation in the Prevention of Myocardial Ischemic Reperfusion Injury: A Systematic Review and Potential Molecular Mechanisms. *Sci. Rep.* **7**, 42386; doi: 10.1038/srep42386 (2017).

**Publisher's note:** Springer Nature remains neutral with regard to jurisdictional claims in published maps and institutional affiliations.

## Supplementary Material

Supplementary Material

## Figures and Tables

**Table 1:** Risk of bias for animal studies, assessed using the SYRCLE^50^ tool.

Study	1	2	3	4	5	6	7	8	9	10
	Selection bias 1	Selection bias 2	Selection bias 3	Performance bias 1	Performance bias 2	Detection bias 1	Detection bias 2	Attrition bias	Reporting bias	Other potential bias
Ad *et al*.^61^	✓	x	x	?	x	x	✓	✓	✓	✓
Biasibetti *et al*.^109^	x	✓	x	?	x	x	x	x	✓	✓
Gatsura *et a*l.^56^	x	x	x	?	x	x	x	x	✓	?
Hentschke *et al*.^71^	x	✓	x	?	x	x	x	x	✓	✓
Keszler *et al*.^55^	?	x	x	?	x	x	x	x	✓	✓
Keszler *et al*.^54^	x	x	x	?	x	x	x	x	?	?
Lohr *et al*.^62^	✓	x	x	?	x	x	x	x	✓	✓
Malinovaskaya *et al*.^111^	?	x	x	?	x	x	x	✓	?	?
Manchini *et al*.^63^	?	✓	x	?	x	x	x	✓	✓	✓
Oron *et al*.^46^	?	x	x	?	x	x	x	✓	✓	✓
Quirk *et al*.^45^	x	✓	x	?	x	x	x	✓	✓	?
Tuby *et al*.^59^	x	x	x	?	x	x	x	x	✓	?
Tuby *et al*.^60^	?	x	x	?	x	x	x	x	✓	✓
Yaakobi *et al*.^58^	?	x	x	?	x	x	x	✓	✓	✓
Yang *et al*.^57^	?	x	x	?	x	x	x	✓	✓	?
Yang *et al*.^51^	?	x	x	?	x	x	x	✓	✓	?
Zhang *et al*.^121^	✓	x	x	?	x	x	x	x	✓	✓

1 ✓ = Adequate randomization; ? = randomized but no details; x = no evidence of randomization.

2 ✓ = Baseline characteristics given; x = baseline characteristics not given.

3 ✓ = Evidence of adequate concealment of groups; x = no evidence of adequate concealment of groups.

4 ✓ = Evidence of random housing of animals; ?  = unknown housing arrangement.

5 ✓ = Evidence of caregivers blinded to intervention; x = no evidence of caregivers blinded to intervention.

6 ✓ = Evidence of random selection for assessment; x = no evidence of random selection for assessment.

7 ✓ = Evidence of assessor blinded; x = no evidence of assessor blinded.

8 ✓ = Explanation of missing animal data; x = no explanation of missing animal data.

9 ✓ = Free of selective reporting based on methods/results; ? = insuive reporting; x = selective reporting.

10 ✓ = Free of other high bias risk; ? = insufficient data to determine risk of other bias.

**Table 2:** Photobiomodulation intervention in models of cardiovascular damage.

Study	Model	Tissue and Histological Findings	Molecular Effects	Intervention Wavelength (nm) Dose (J) Timing
***animal models***				
Ad *et al*.^61^	Rats (n = 41)	↓ infarct size (65% compared to control)		trans-thoracic Laser 804 nm 4.5 J 60 sec post-conditioning (10 min; 3days)
Biasibetti *et al*.^109^	Rats (n = 49)	No change in haemodynamic variables ↓ (non-significant) infarct size	↓ SOD activity ↓ DCFH ↓ GPX	leg muscle Laser 660 nm 2.1 J 10 sec 14.7 J 73.5 sec DNA damage post-conditioning (4 weeks)
Gatsura *et al*.^56^	Rats (n = 42)	↓ infarct size	↑ SOD ↓ Hb affinity for O2	trans-thoracic Laser 660 nm 0.138 J 60 sec post-conditioning (4 hour)
Hentschke *et al*.^71^	Rats (n = 9)	↓ infarct size (non-significant)	↓IL-6 ↑IL-6 (21 J) ↓CPK ↓ TNF-α (3 J & 21 J) ↑ IL-10 (3 J)	leg muscle Laser 660 nm 14.7 J 73.5 sec; 2.1J 10 secpost-conditioning (4 weeks)
Keszler *et al*.^55^	mice	↓ infarct size	↑ NO from HbNO & MbNO, independent of NOS	myocardium LED 660 nm 51 J 60 sec during surgery
Keszler *et al*.^54^ mice	mice	↓ infarct size	↑ NO from HbNO & MbNO, independent of NOS ↑ NADH	myocardium LED 660 nm 8.5 J 60 sec during surgery
Lohr *et al*.^62^	rabbit	↓ infarct size with 11 J; (0.5 J ineffective) Synergistic effect with nitrite & blocking effect of nitric oxide scavenger	↑ HbNO conversion to metHb (4x) Increased nitrite reductase activity of deoxyHb, ↓MbNO signal in ischemic zone by ~60%.	myocardium Laser 670 nm 14 J 41 sec pre and post-conditioning and during surgery
Malinovaskaya *et al*.^111^	Rat (n = 91)	↓ mortality with LED (better) & laser	↓ LPO LED (better) and laser ↑ SOD activity (LED) ↓ SOD activity (laser)	myocardium Laser & LED 640 nm 5 J post-conditioning (after reperfusion)
Manchini *et al*.^63^	Rat (n = 82)	↓ acute myocardium inflammation ↓ infarct size ↓ no. of large infarcts improved post MI left ventricle dysfunction	↑IL-1β and IL-6 (3 days after MI) ↑ plasma kallikrein ↑ Kinin B2 receptor gene expression ↓ kinin B1, ACE gene expression ↑ Mas receptor gene expression ↓ iNOS gene plasma ↑ nitrite and nitrate (NOx)	myocardium Laser660 nm 1.1 J 60 sec post-conditioning (after reperfusion)
Oron *et al*.^53^	Dog (n = 50) rat (n = 26)	↓ cardiac infarct size ↓ dense collagen, Less disorganised cardiomyocytes ↓ mitochondrial damage (21%) ↓ scarring	↑ (7.3x) desmin-expressing structures ↓ (22%) release of troponin-T	myocardium Laser (dogs) 803 nm trans-thoracic Laser (rats) 1.08 J 180 sec post-conditioning
Quirk *et al*.^52^	Rat (n = 75)	Safe application ↓ infarct size by up to 40% no change in hemodynamic data	↓Troponin post PBM CK and LDH did not change	myocardium LED 670 nm 26 J 60 sec during reperfusion
Tuby *et al*.^59^	Rat (n = 147)	↓ Infarct size 64% (5 mW) 69% (12 mW) ↑ cardioprotection & angiogenesis.	↑ VEGF (24–48 hrs) no difference (72 hrs) Significant. angiogenesis (5, 12 mW/cm^2^) ↑ iNOS, (peak 2–5 days)	myocardium + trans-thoracic Laser 804 nm 0.6 J, 1.44 J, 2.04 J 2 m pre-& post-conditioning
Tuby *et al*.^60^	Rat (n = 72)	↓ MI cardiomyocyte scarring ↓ infarct size 31% 76% when applied to tibia	Hypothesised autologous stem cell recruitment.	myocardium + tibia bone Laser804 nm 1 J 100 secpost-conditioning
Yaakobi *et al*.^58^	Rat (n = 65)	↓ necrotic & scar tissue ↓ infarct size (14,21,45 days), ↓ Left ventricular dilatation ↑ endothelial cell and 3.1x vessel proliferation	↑ HSP70i (2.2x)	trans-thoracic Laser 804 nm 0.27 J 60 sec post-conditioning (3 days)
Yang *et al*.^119^	Rat (n = 94)	↓ infarct size protective effect on injured cardiac myocardium. No change to heart function	↑ cytokines: AB, IL10, TIMP-1, VEGF, GM-CSF, IL4; ↓ CiNC-3, ↓ Fractalkine to control levels by week 2	myocardium Laser 635 nm 1 J 150 sec post-conditioning (after reperfusion)
Yang *et al*.^51^	Rat (n = 120)	↓ Infarct size ↑ left ventricle wall thickness, ↑ attenuation of collagen fibres	↑ MDA ↓ SOD	myocardium Laser 635 nm 0.96 J 150 sec post-conditioning (3 weeks)
Zhang *et al*.^121^	Rat (n = 190)	Improved angiogenesis and LV function.	↑ VEGF (1 hr & 1 day) ↑ SOD; ↑ GRP78 mRNA ↓ MDA	myocardium Laser 635 nm 0.96 J 150 sec pre-conditioning
***in vitro tissue and cells***				
Gavish *et al*.^70^	Porcine aortic cells	↑ smooth muscle cell proliferation (15%, 23%,11% at 24, 48, 72 hrs). ↑ type 1,3 collagen	↑ activity TIMP-2 ↑ MMP-1&2	Laser 780 nm 2 J 540 sec pre conditioning
Monich *et al*.^112^	isolated rat hearts	Restored myocardial contractility	↑ SOD ↓ Ca^2+^ in region indicating ↑ flow	Laser 660 nm 1.03 J 60 sec during reperfusion
Plass *et al*.^122^	human LAD	Significant ↑ photo-relaxation of left anterior descending vessels. 73% of the maximal obtainable effect by an endothelial vasodialator		Laser 660 nm 16 J 180 sec post-conditioning
Tuby *et al*.^69^	cardiac stem cells	↓ MI cardiomyocyte scarring ↑ mesenchymal stem cells. ↑ cardiac stem cells		Laser 804 nm 1 J 20 sec; 3 J 60 sec pre-conditioning
Zhu *et al*.^85^	isolated rat hearts	↑ cardiac functionality.	↑ NO (18 hrs) ↑ ATP	Laser 660 nm pre: 16.8 J 420 sec post: 36 J 420 sec pre-conditioning & during reperfusion
***clinical trial***				
Khoo *et al*.^78^	CABG (n = 64)	↑ cardiac tissue repair post-operatively	↓ CPK, CPK-MB. Slight ↑ LDH. ↓ WBC’s, ↓ Lymphocytes ↓ Neutrophils.	trans-thoracic Laser 810 nm 6 J post-conditioning
