# Supplementary material for: A Role for Photobiomodulation in the Prevention of Myocardial Ischemic Reperfusion Injury: A Systematic Review and Potential Molecular Mechanisms
Source: Sci Rep. 2017 Feb 9;7:42386. doi: 10.1038/srep42386 (PMC5299427; doi:10.1038/srep42386)
Supplement: Supplementary Material [file srep42386-s1.pdf]

A Role for Photobiomodulation in the Prevention of Myocardial Ischemic-Reperfusion Injury: A Systematic Review and Potential Molecular Mechanisms

Ann Liebert, Andrew Krause, Neil Goonetilleke, Brian Bicknell, Hosen Kiat

Supplementary Table S1. *Search results from databases*

Database searched	Results
Scopus	125
Pubmed	135
Ovid/OvidMedline	8
Web of Science	128
Total search results	396
Additional papers identified from references	4

Supplementary Table S2. *Eligibility criteria:* articles must meet *all* of the following criteria

1	Intervention which studied PBM, limited to wavelengths between 600 – 1000 nm
2	Studies reported a minimum of wavelength, power output and dose intervention parameters, or could be calculated from data in the article.
3	Experimental model or clinical investigation was representative of Myocardial Ischemic Reperfusion injury
4	All relevant quantifiable outcome measures were compared to control (no light) treatment.
5	Up to date articles, defined as published after 1995.
6	Articles available in English language.
7	Studies reported a minimum of one outcome measure, related either to molecular signalling or histological change.

Supplementary Table S3. *Articles excluded from qualitative analysis due to exclusion criteria*

Author	Experimental Model	Result	Reason for exclusion
Tuby et al. 2008[1]	Transplantation of irradiated donor mesenchymal bone marrow stem cells into infarcted rat heart	LLLT can significantly increase survival and/or proliferation of myocardial tissue.	cell culture was exposed to 5-bromo-2'deoxyuridine every two days for two weeks prior to intervention.
De Scheerder *et al* 2000[2]	Intravascular irradiation with stenosis in humans with coronary heart disease	decreased post stent restenosis rate<br>No complications seen.	Stenting procedure
De Scheerder *et al*. 2001[3]	Intravascular irradiation with stenosis in porcine cells	Reduced post stent restenosis<br>decreased neointimal hyperplasia	Stenting procedure
De Scheerder *et al*. 2001[4]	Intravascular irradiation with stenosis in humans with coronary heart disease	decreased restenosis rates post cardiac stenting<br>decreased late luminal loss<br>decreased late loss index	Stenting procedure
Derkacz et al. 2003[5]	Intravascular irradiation with stenosis in humans with coronary heart disease	no indication of restenosis and enhance exercise tolerance till onset of angina.	Stenting procedure
Derkacz *et al*. 2004[6]	Intravascular irradiation with stenosis in humans with coronary heart disease	no side effects<br>decreased restenosis rates<br>long-term no restenosis in PBM group.	Stenting procedure
Derkacz *et al*. 2010[7]	Intravascular irradiation with stenosis in humans with coronary heart disease	decreased LLLI and significantly less loss of vascular lumen at 12 month follow-up	Stenting procedure
Derkacz *et al*. 2013[8]	Intravascular irradiation with stenosis in humans with coronary heart disease	decreased loss of vascular lumen (LLL) and LLLI compared to control<br>decreased IL-1β, IL-6, IL-10	Stenting procedure
Kimura et al. 2013[9]	Intravascular irradiation of cavotricuspid isthmus in canines	LLLT comparable to radiofrequency ablation procedures	experimental model not representative of cardiovascular pathology
Pinto et al. 2009[10]	Laser on Saphenectomy dehiscence in humans after CABG	advanced wound closure, enhanced granulation tissue formation, reduced fibrin	experimental model not representative of cardiovascular pathology
Salem et al. 2006[11]	Intravascular irradiation in humans with angina	reduced need for medication and further intervention, no significant change in ejection fraction	no control and experimental model not representative of cardiovascular pathology
Tomimura et al. 2014[12]	intravascular irradiation on spontaneously hypertensive rats	Reduced blood pressure at long term follow up	experimental model not representative of cardiovascular pathology
Zhao et al. 2006[13]	Effect of intravascular irradiation on induced bradycardia in rabbits	no significant recovery from bradycardia with 650nm laser. CO2 combined laser unit effective at enhancing recovery from bradycardia.	experimental model not representative of cardiovascular pathology

BReferences

1 Tuby, H., Maltz, L. & Oron, U. Implantation of low-level laser irradiated mesenchymal stem cells into the infarcted rat heart is associated with reduction in infarct size and enhanced angiogenesis. *Photomedicine and laser surgery* **27**, 227-233 (2009).

2 De Scheerder, I. *et al.* Long-term follow-up after coronary stenting and intravascular red laser therapy. *The American journal of cardiology* **86**, 927-930 (2000).

3 De Scheerder, I. K. *et al.* Optimal dosing of intravascular low-power red laser light as an adjunct to coronary stent implantation: insights from a porcine coronary stent model. *Journal of clinical laser medicine & surgery* **19**, 261-265 (2001).

4 De Scheerder, I. K. *et al.* Intravascular low-power laser irradiation after coronary stenting: Long-term follow-up. *Lasers in surgery and medicine* **28**, 212-215 (2001).

5 Derkacz, A. *et al.* Photostimulation of coronary arteries with low power laser radiation: preliminary results for a new method in invasive cardiology therapy. *Medical Science Monitor* **9**, CR335-CR339 (2003).

6 Derkacz, A., Bialy, D., Protasiewicz, M., Beres-Pawlik, E. M. & Abramski, K. M. in *Proc. SPIE 5505, Optical methods, sensors, image processing, and visualization in medicine.* 51-55 (2004).

7 Derkacz, A., Protasiewicz, M., Poreba, R., Szuba, A. & Andrzejak, R. Usefulness of intravascular low-power laser illumination in preventing restenosis after percutaneous coronary intervention. *American journal of cardiology* **106**, 1113-1117, doi:10.1016/j.amjcard.2010.06.017 (2010).

8 Derkacz, A., Protasiewicz, M., Poreba, R., Doroszko, A. & Andrzejak, R. Effect of the intravascular low energy laser illumination during percutaneous coronary intervention on the inflammatory process in vascular wall. *Lasers in medicine and science* **28**, 763-768, doi:10.1007/s10103-012-1142-z (2013).

9 Kimura, T. *et al.* Nonthermal cardiac catheter ablation using photodynamic therapy. *Circulation: Arrhythmia and Electrophysiology* **6**, 1025-1031 (2013).

10 Pinto, N. C., Pereira, M. H. C., Stolf, N. A. G. & Chavantes, M. C. Low level laser therapy in acute dehiscence saphenectomy: therapeutic proposal. *Revista Brasileira de Cirurgia Cardiovascular* **24**, 88-91 (2009).

11 Salem, M., Rotevatn, S. & Nordrehaug, J. E. Long-term results following percutaneous myocardial laser therapy. *Coronary artery disease* **17**, 385-390 (2006).

12 Tomimura, S. *et al.* Hemodynamic Effect of Laser Therapy in Spontaneously Hypertensive Rats. *Arquivos brasileiros de cardiologia* **103**, 161-164 (2014).

13 Zhao, L. *et al.* Effect of different LLLT on pituitrin-induced bradycardia in the rabbit. *Lasers Med Sci* **21**, 61-66 (2006).
